# Editorial: Evolutionary Theory: Fringe or Central to Psychological Science

**DOI:** 10.3389/fpsyg.2016.00777

**Published:** 2016-05-24

**Authors:** Danielle Sulikowski

**Affiliations:** School of Psychology, Charles Sturt UniversityBathurst, NSW, Australia

**Keywords:** e-cognition, evolutionary psychology, massive modularity, developmental niche construction, computational theory of mind, developmental plasticity, biocultural evolution

The computational theory of mind, which views the brain as an information processor that operates on cognitive representations, is central to modern cognitive psychology and is the dominant perspective from which brain function is conceptualized and studied. Evolutionary Psychology (EP) is the application of evolutionary theory to understanding human behavior and cognition. Unlike other core Psychology topic areas (such as Personality, Learning, or Developmental Psychology), however, EP is not defined by the subset of psychological phenomena it seeks to describe and understand. It is instead defined by a specific meta-theoretical perspective, from which it seeks to (potentially) explain all psychological phenomena. The central question posed by this volume is whether this over-arching nature provides an opportunity for evolutionary approaches to offer an alternative meta-theoretical perspective to the information processing/representational view of brain function and behavior.

Readers of this volume will notice a sharp demarcation between descriptions of traditional Evolutionary Psychology, which several authors (Barret et al.; Stotz; Stulp et al.) have presented as indistinguishable from the information processing approach, and newer conceptualizations of EP. Indeed one of the major themes running through several of the contributions (Burke; Barret et al.; Stephen; Stotz; Stulp et al.) concerns the appropriate conceptualization of EP itself, with the Santa Barbara school of massive modularity (made famous by John Tooby and Leda Cosmides) receiving the most scrutiny. As Barret et al. and Stotz describe, early conceptualizations of EP embraced the notion of massive modularity of mind. Individual modules were presumed to act as evolved computers, sensitive to domain specific information and processing it in adaptive ways. Framed in this manner, EP fits well within even a very strict definition of a computational theory of mind and could hardly be seen as the source of an alternative meta-theoretical approach to understanding brain and behavior.

It may not be appropriate, however, to view either the computational theory of mind or the field of EP so narrowly. As Klasios argues, many evolutionary psychologists adopt a more generic notion of computation, one that commits more to the abstract representation and manipulation of information, rather than to digital computation in its literal sense (although see also Bryant). EP too, is no longer wed to notions of massive modularity (Stephen), with the majority of research in the field motivated by consideration of first principles of evolutionary theory and is neither constrained nor informed by assumptions of massive modularity or domain specific mechanisms (Burke). With these considerations in mind, Klasios and Bryant both argue that computation is still the most profitable account of the mind and is able to accommodate both evolutionary and e-cognition (extended, embodied approaches which place emphasis on the role played by the whole organism and its environment in the decision-making process, rather than simply the brain) perspectives, that favor notions of neural adaptations that are “complex, widely distributed, and highly diffuse” (Klasios) over the more strictly isolated mental modules supposed by massive modularity.

Burke further argues that commitments to massive modularity, or to either a computational, direct, or e-cognition view of the brain, are unnecessary for evolutionary theory to become the foundational theory of psychological science. Presenting a series of six reasons for the current failure of evolutionary theory to inform most research within psychological science, Burke (with supporting arguments given by Jonason and Dane, and Stephen), suggests that a mixture of misunderstandings about the field of EP coupled with motivated opposition and misguided skepticism are to blame.

If Burke’s assessment is accurate, such barriers may only be overcome by a concerted effort to unite EP with Behavioral Ecology and Evolutionary Biology. Stotz proposes one such unity with her Extended Evolutionary Psychology. Combining evolutionary theories concerning genetic, epigenetic, behavioral, and cultural systems of inheritance, developmental plasticity and niche construction, with e-cognition, Stotz outlines a truly integrative EP. Stotz’ Extended Evolutionary Psychology draws on complex mechanisms of inheritance to help understand the evolution of psychological traits. But it also sees investigations of e-cognition informing theories of niche construction and transgenerational developmental plasticity. Thus, the integration of evolutionary theory with psychology provides reciprocated benefits to both fields.

Barrett et al.; Barrett et al. and Stulp et al. argue for an Extended Mind Hypothesis. The Extended Mind Hypothesis sits within an evolutionarily informed framework, but places much emphasis on the sociocultural nature of human psychology and the external resources (cultural and technological artifacts) that form part of the modern human cognitive system. The Extended Mind Hypothesis offers the various forms of e-cognition, rather than EP, as the appropriate meta-theoretical perspective to succeed the computational theory of mind. In arguments that mirror those presented by Burke, however, Stephen et al. argue that while e-cognition represents an interesting alternative to more traditional proximal explanations of behavior (such as computational theory of mind), behavior must still be examined through a sophisticated evolutionary lens if an ultimate understanding is to be reached.

Newer conceptualizations of EP are uncommitted to notions of massive modularity, look beyond the Pleistocene for the selection pressures that have shaped psychological mechanisms and incorporate developmental and cultural impacts into theories concerning the evolved functions of psychological mechanisms. It is clear however, that the massive modularity roots of modern EP still influence how many, including both advocates and critics, view the field. One message that is clear from the works presented in this volume, is that EP must mature and free itself of many of its early assumptions and assertions (as seems to be currently happening empirically, if not yet theoretically, Burke). Only if this occurs, will EP be placed to properly integrate with Evolutionary Biology and be in a position to cement evolutionary theory as a unifying meta-theory for Psychological Science. Whether such a New Evolutionary Psychology should incorporate computational theories of mind or reject these in favor of the newer e-cognition perspectives is an empirical question and not one whose answer needs to be decided before the weight of evidence has settled in either court (Stephen).

## Author contributions

The author confirms being the sole contributor of this work and approved it for publication.

### Conflict of interest statement

The author declares that the research was conducted in the absence of any commercial or financial relationships that could be construed as a potential conflict of interest.

